# Prognostic value of CDCA3 in kidney renal papillary cell carcinoma

**DOI:** 10.18632/aging.203767

**Published:** 2021-12-14

**Authors:** Hao Li, Mi Li, Caihong Yang, Fengjing Guo, Sisi Deng, Lixi Li, Tian Ma, Jiyuan Yan, Hua Wu, Xiaojuan Li

**Affiliations:** 1 Department of Orthopedics, Tongji Hospital, Tongji Medical College, Huazhong University of Science and Technology, Wuhan 430030, Hubei, China; 2 Department of Nephrology, Tongji Hospital, Tongji Medical College, Huazhong University of Science and Technology, Wuhan 430030, Hubei, China; 3 Cancer Center, Union Hospital, Tongji Medical College, Huazhong University of Science and Technology, Wuhan 430030, Hubei, China

**Keywords:** cell division cycle associated 3, kidney renal papillary cell carcinoma, prognostic values, differential expression, immune cell infiltration

## Abstract

Kidney renal papillary cell carcinoma (KIRP) is a type of low-grade malignant renal cell carcinoma. Huge challenges remain in the treatment of KIRP. Cell division cycle associated 3 (CDCA3) participates in human physiological and pathological processes. However, its role in KIRP has not been established. Here, we evaluated the prognostic value of CDCA3 in KIRP using a comprehensive bioinformatics approach. Data for CDCA3 expression in KIRP were obtained from online database. Different expression genes between high and low CDCA3 expression groups were identified and evaluated by performing Gene ontology and Kyoto Encyclopedia of Genes and Genomes pathway enrichment analyses. A gene set enrichment analysis was performed to elucidate the function and pathway differences between the different. Differences in immune cell infiltration between low and high CDCA3 expression groups were analyzed by a single-sample GSEA method for immune cells. A protein-protein interaction network was generated and hub genes were identified. UALCAN was used to analyze associations between the mRNA expression levels of CDCA3 in KIRP tissues with clinicopathologic parameters. The diagnostic efficacy of CDCA3 for KIRP was analyzed by ROC analysis. Logistic regression was used to analyze relationships between the clinicopathological characteristics and CDCA3 expression. Our results indicated that high CDCA3 mRNA expression is significantly associated with some clinicopathologic parameters in KIRP patients High CDCA3 mRNA expression associated with a shorter overall survival, progression-free interval, and disease-special survival. Taken together, CDCA3 is a potential target for the development of anti-KIRP therapeutics and is an efficient prognostic marker.

## INTRODUCTION

Renal cell carcinoma (RCC) is one of the most common malignant tumors of the urinary system, and its morbidity and mortality are on the rise worldwide [1]. Kidney renal papillary cell carcinoma (KIRP), also known as papillary renal cell carcinoma (PRCC), is a type of low-grade malignancy that originates from renal tubular epithelial cells [2], accounting for about 10-20% of RCC cases [3–6]. KIRP grows slowly and has a better prognosis than those of other types of RCC.

Currently, imaging examinations such as ultrasound, computed tomography (CT), and magnetic resonance imaging (MRI) are used to diagnose KIRP, however, these approaches do not show sufficient specificity [7]. Therefore, the pathological examination remains the gold standard [8, 9]. Nephrectomy and nephron-sparing surgery are still the main treatments for KIRP. Chemotherapy and targeted drugs exert certain effects in advanced metastatic KIRP, however, the efficacy of these approaches remains controversial [3, 4]. In addition, the cost of the KIRP diagnosis and treatment imposes a heavy burden to individuals and society.

Although KIRP has a low rates of metastasis and recurrence [10, 11], prognosis, especially for patients with advanced disease, is very poor due to occurrence of distant metastasis [12]. Owing to the lack of clinical symptoms, KIRP is usually found on physical examination. A high tumor volume is associated with cystic changes, necrosis, bleeding, and calcification [13]. Therefore, the identification of credible predictors related to the stage and prognosis of KIRP will help to provide new targets for treatment, diagnosis, and prognostic evaluation. Various biomarkers associated with KIRP progression and prognosis have been reported [14–16], however, their credibility remains controversial.

Gene encoding CDCA3 is located on chromosome 12p12 and the protein is composed of 268 amino acids with a molecular weight of 29 kDa. CDCA3 contributes to human physiological and pathological processes by regulating various downstream cytokines. Studies have shown that CDCA3 plays an important role in the development of various tumors [17–19]. However, little is known about the role of CDCA3 in the KIRP development.

In this study, we addressed this issue by identifying the transcriptional expression patterns of CDCA3 based on The Cancer Genome Atlas (TCGA) database and the Genotype-Tissue Expression (GTEx) database. We further evaluated Gene Ontology (GO) functions and Kyoto Encyclopedia of Genes and Genomes (KEGG) pathways of CDCA3 related to CDCA3 and associated differential expression genes (DEGs) in KIRP. Furthermore, we performed a gene set enrichment analysis (GSEA), immune infiltration analysis, protein-protein interaction (PPI) network analysis, clinicopathologic analysis, and analyzed the prognostic value of CDCA3 in KIRP. Our study clarify the biological functionality and prognostic value of CDCA3, which is expected to be beneficial for the diagnosis and treatment of KIRP.

## MATERIALS AND METHODS

### Differential expression of CDCA3

The TCGA database was used to investigate CDCA3 expression in patients with KIRP and analyze the association between expression levels and the prognosis. In total, 320 samples were selected as the TCGA cohort including 288 KIRP samples and 32 normal samples. Level 3 high-throughput RNA-sequencing data and corresponding clinical information data were downloaded from the KIRP project of the TCGA GDC data portal. RNAseq data in FPKM (fragments per kilobase per million) format were converted into TPM (transcripts per million reads) format for comparisons of CDCA3 expression levels between samples. The Wilcoxon rank-sum test was used to compare the gene expression levels of CDCA3 in 32 normal samples and 288 KIRP samples and between 31 KIRP samples and the paired adjacent normal tissues were compared. Results with *P* < 0.001 were considered statically significant.

RNAseq data were downloaded in TPM format from UCSC XENA (https://xenabrowser.net/datapages/), and these data were processed in a unified way through the Toil process [20] from TCGA and GTEx database. The expression of CDCA3 in normal samples of the GTEx database and TCGA database was compared with corresponding 33 types of cancer samples including KIRP in TCGA by Wilcoxon rank-sum test. Results with *P* < 0.001 were considered statically significant.

### DEGs associated with CDCA3 in KIRP

According to the median expression levels of CDCA3 (TPM values) in KIRP from TCGA database, all KIRP samples were divided into two groups: CDCA3-high expression group and CDCA3-low expression group. The DESeq2 package [21] was used to analyze the DEGs correlated with CDCA3 expression in KIRP from the TCGA database by using RNA-seq count data downloaded from the GDC data portal.

### GO and KEGG pathway enrichment analyses

Metascape (http://metascape.org) was used to analyze the functional and pathway enrichment of DEGs and generate PPI networks associated with CDCA3 alterations in KIRP. GO and KEGG pathways enrichment was analyzed using Metascape [22]. *P* < 0.01, a minimum count of 3, and the enrichment factor > 1.5 were thresholds for statistical significance.

### Gene set enrichment analysis (GSEA)

GSEA [23] was performed using R package clusterProfiler (3.8.0) to elucidate the significant functional and pathway differences between the CDCA3-low expression group and the CDCA3-high expression group [24]. The h.all.v7.0.symbols.gmt file in MSigDB Collections was selected as the reference gene collection. The number of gene set permutations was 1,000 for each analysis. NES absolute value >=1, adjusted *P*-value < 0.05, and FDR < 0.25 were considered to be statistically significant.

### Immune cell infiltration analysis by ssGSEA

Immune cell infiltration analysis was analyzed by a ssGSEA for 24 types of immune cells in tumor samples [25]. These 24 types of immune cells comprised macrophages, neutrophils, B cells, cytotoxic cells, T cells, CD8+ T cells, NK cells, NK CD56bright cells, NK CD56dim cells, mast cells, eosinophils, dendritic cells (DCs), activated DCs (aDCs), plasmacytoid DCs (pDCs), immature DCs (iDCs), T helper cells (Th), Th1 cells, Th2 cells, Th7 cells, Regulatory T cells (Treg), T gamma delta (Tgd), T central memory (Tcm), T effector memory (Tem) and T follicular helper (Tfh). The correlations between CDCA3 expression and these immune cell frequencies were analyzed by Spearman correlation coefficients, and the infiltration of immune cells was compares between the CDCA3-low group and CDCA3-high group by the Wilcoxon rank-sum test.

### PPI network analysis

The STRING database (Search Tool for the Retrieval of Interacting Genes) (http://string-db.org) was used to analyze the functional interactions between proteins [26]. The PPI networks were constructed using Cytoscape based on STRING with a threshold for interaction score of 0.7. The most significant module in the PPI network was identified by MCODE (Molecular Complex Detection) embedded in Cytoscape to identify densely connected regions. The criteria for selection were as follows: degree cut-off =2, node score cut-off = 0.2, Max depth = 100 and k-score = 2.

### Clinicopathological analysis of CDCA3 in KIRP

UALCAN was used to analyze the associations between the mRNA expression level of CDCA3 in KIRP tissues with their clinicopathologic parameters, such as clinical stage, patient’s gender, race, age, smoking status, serum calcium, hemoglobin, laterality and MET status. The results were obtained directly by selecting the clinicopathological grouping options integrated into the UALCAN database. Only the tumor group could be divided into different clinicopathological groups. *P* < 0.05 indicated significance.

### Receiver operating characteristic (ROC) curve

The AUC of the ROC curve was generated to evaluate the predictive value of the gene. AUC values closer to 1.0 indicated a better diagnosis, 0.5 ~ 0.7 indicated a low predictive value, 0.7 ~ 0.9 indicated moderate predictive accuracy, and > 0.9 indicated a high accuracy. The abscissa was the false positive rate (FPR), and the ordinate was the true positive rate (TPR).

### Survival analysis

The prognostic value of the CDCA3 mRNA expression level in KIRP was analyzed using the survminer package of R. Based on the median values of CDCA3 expression (TPM), patients with KIRP were divided into CDCA3-low expression group and CDCA3-high expression group. Results with *P* < 0.05 were considered statically significant.

### Ethics statement

As all data used in this study were obtained from the TCGA database. Hence, ethics approval and informed consent were not required. Our study was performed in accordance with the publication guidelines of TCGA.

### Statistical analyses

All statistical analyses and the generation of plots were performed using R (v.3.5.1). The Wilcoxon rank-sum test and Wilcoxon signed-rank test were used to compare the expression of CDCA3 in unpaired samples and paired samples, respectively. The Kruskal-Wallis test, Wilcoxon signed-rank test, and logistic regression were used to evaluate the relationships between clinical-pathologic features and CDCA3 expression. Cox regression analyses and the Kaplan-Meier method were used to evaluate prognostic factors. A multivariate Cox analysis was used to evaluate the impact of CDCA3 expression on survival along with other clinical traits.

## RESULTS

### Overexpression of CDCA3 in patients with KIRP

**Figure 1:**
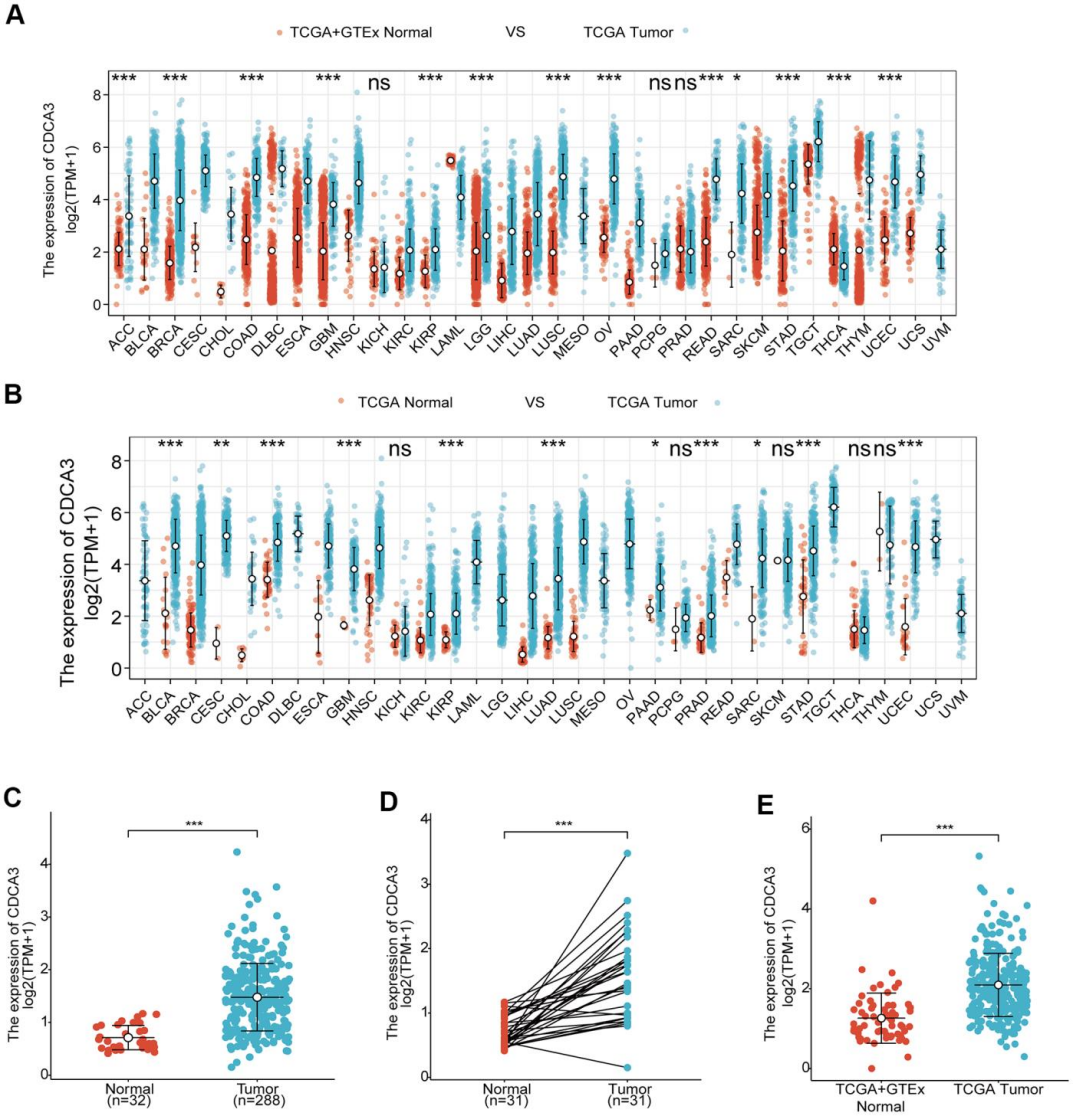
**Overexpression of CDCA3 in patients with KIRP.** (**A**) CDCA3 expression in normal samples from the GTEx database and the TCGA and 33 tumor samples in TCGA. ns, *p* ≥ 0.05; *, *p* < 0.05; **, *p*< 0.01; ***, *p* < 0.001. (**B**) CDCA3 expression in 33 tumor samples in TCGA and paired paracancerous tissues in TCGA. ns, *p* ≥ 0.05; *, *p* < 0.05; **, *p* < 0.01; ***, *p* < 0.001. (**C**) CDCA3 mRNA expression level in 288 KIRP samples and 32 normal samples. *** *p* < 0.001. (**D**) CDCA3 mRNA expression in KIRP tissues and in paired paracancerous normal samples. *** *p* < 0.001. (**E**) CDCA3 mRNA expression in normal samples and KIRP from the GTEx database and TCGA. *** *p* < 0.001.

We analyzed CDCA3 expression in normal samples from the GTEx database and the TCGA and 33 tumor samples in TCGA. CDCA3 expression was significantly up-regulated in bladder urothelial carcinoma, cervical squamous cell carcinoma and adenocarcinoma, KIRP, KIRC, and other cancer types (Figure 1A). An analysis of various tumors and the paired paracancerous tissues in TCGA showed that the expression of CDCA3 in bladder urothelial carcinoma, KIRP, hepatocellular carcinoma and other cancers was significantly higher than those in corresponding paracancerous tissues (Figure 1B).

To detect the differences in the CDCA3 mRNA expression level between tumor and non-cancerous tissues, RNAseq data for 288 KIRP samples and 32 normal samples were analyzed. As was shown in Figure 1C, CDCA3 mRNA expression level were significantly higher in KIRP samples than in normal tissues. The up-regulation of CDCA3 mRNA expression was also observed in KIRP tissues compared to that in paired paracancerous normal samples (Figure 1D). Furthermore, based on expression data for normal samples from the GTEx database and TCGA as well as KIRP samples from TCGA, CDCA3 was significantly overexpressed in KIRP (Figure 1E).

These results indicated that the expression of CDCA3 is up-regulated in various types of tumor tissues, including KIRP, in which it is significantly overexpressed compared with levels in normal kidney tissues or paired paracancerous normal samples.

### DEGs associated with CDCA3 in KIRP

**Figure 2:**
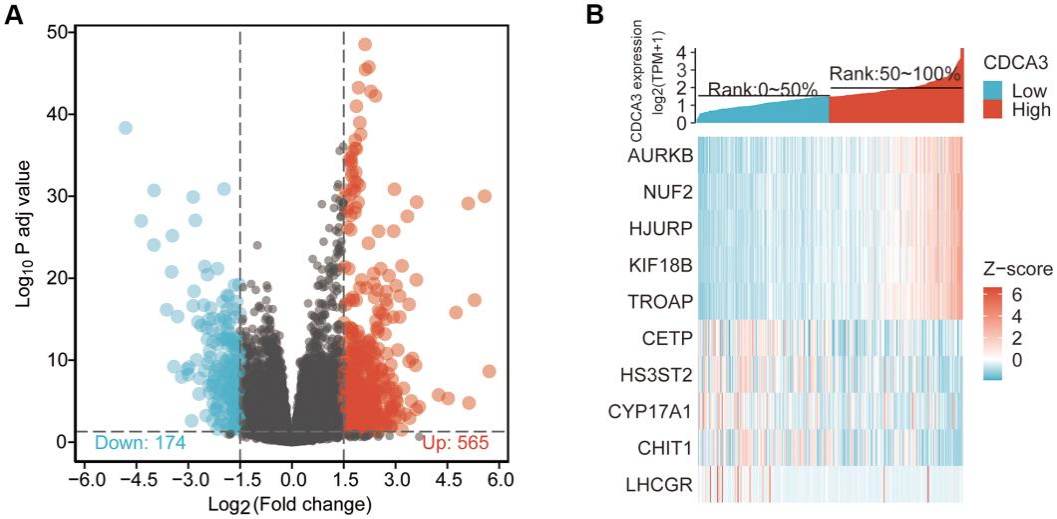
**DEGs associated with CDCA3 in KIRP.** (**A**) The results of the DEGs analysis with a volcano graph. |logFC| > 1.5 and *p*adj < 0.05. (**B**) The results of the most distinctly DEGs with a heat map.

We identified DEGs or co-expressed genes associated with CDCA3 in KIRP by identifying genes that differed in expression between the groups with high and low CDCA3exression. We detected 739 DEGs with |logFC |> 1.5 and *p*adj < 0.05 between groups. A volcano graph was generated to visualize the results of the DEGs analysis. Among the DEGs, 565 had logFC > 1.5 and *p*adj < 0.05, and 174 had logFC < -1.5 and *p*adj < 0.05 (Figure 2A). As shown in Figure 2B, the expression level of *AURKB, NUF2, HJURP, KIF18B* and *TROAP* were significantly up-regulated in high CDCA3 expression group compared with the low CDCA3 expression group, while the expression level of *CETP, HS3ST2, CYP17A1, CHIT1* and *LHCGR* were significantly down-regulated in the CDCA3 high-expression group.

### GO and KEGG pathway enrichment analyses

**Figure 3:**
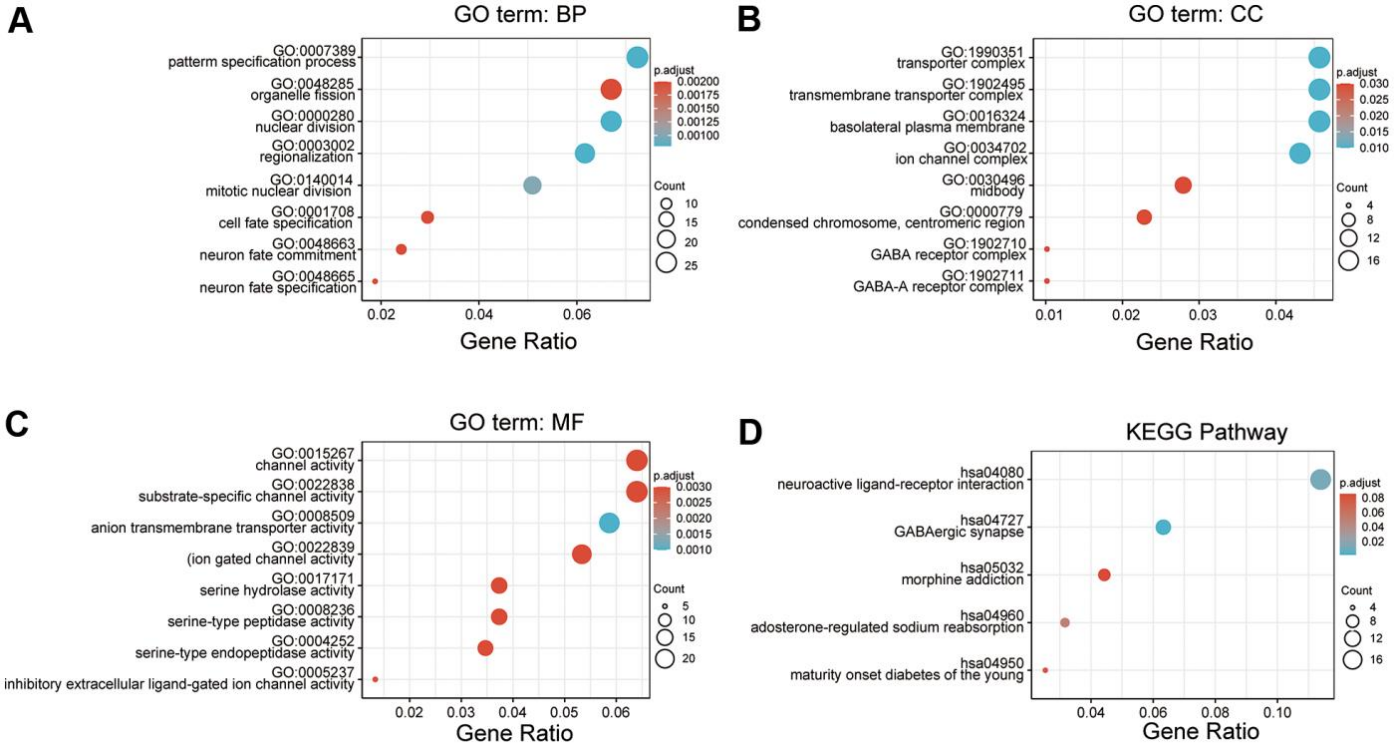
**GO and KEGG pathway enrichment analyses.** (**A**–**C**) GO enrichment analyses of DEGs associated with CDCA3. (**A**) Cellular component; (**B**) Biological processes; (**C**) Molecular functions (**D**) KEGG enrichment analyses of DEGs associated with CDCA3.

Functional and pathway enrichment analyses of DEGs associated with CDCA3 were analyzed using Metascape. Various biological processes, such as GO: 0007389 (pattern specification process), GO: 0048285 (organelle fission), GO: 0000280 (nuclear division), GO: 0003002 (regionalization), GO: 0140014 (mitotic nuclear division), GO0001708 (cell fate specification), GO: 0048663 (neuron fate commitment) and GO: 0048665 (neuron fate specification) were significantly associated with alterations in CDCA3 expression (Figure 3A). Additionally, genes associated with CDCA3 were enriched for various cellular components, including GO: 1990351 (transporter complex), GO: 1902495 (transmembrane transporter complex), GO: 0016324 (basolateral plasma membrane), GO: 0034702 (ion channel complex), GO: 0030496 (midbody), GO: 000779 (condensed chromosome, centromeric region), GO: 1902710 (GABA receptor complex) and GO: 1902711 (GABA-A receptor complex) were remarkably regulated by the CDCA3 in KIRP (Figure 3B). CDCA3 also prominently affected the molecular functions (Figure 3C), such as GO:0015267 (channel activity), GO:0022838 (substrate-specific channel activity), GO:0008509 (anion transmembrane transporter activity), GO:0022839 (ion gated channel activity), GO:0017171 (serine hydrolase activity), GO:0008236 (serine-type peptidase activity), GO:0004252 (serine-type endopeptidase activity), GO:0005237 (inhibitory extracellular ligand-gated ion channel activity).

In a KEGG analysis, these pathways including hsa04080 (Neuroactive ligand-receptor interaction), hsa04727 (GABAergic synapse), hsa05032 (Morphine addiction), hsa04960 (Aldosterone-regulated sodium reabsorption) and hsa04950 (Maturity onset diabetes of the young) pathways associated with CDCA3 function in KIRP (Figure 3D).

### CDCA3-related signaling pathways based on GSEA

**Figure 4:**
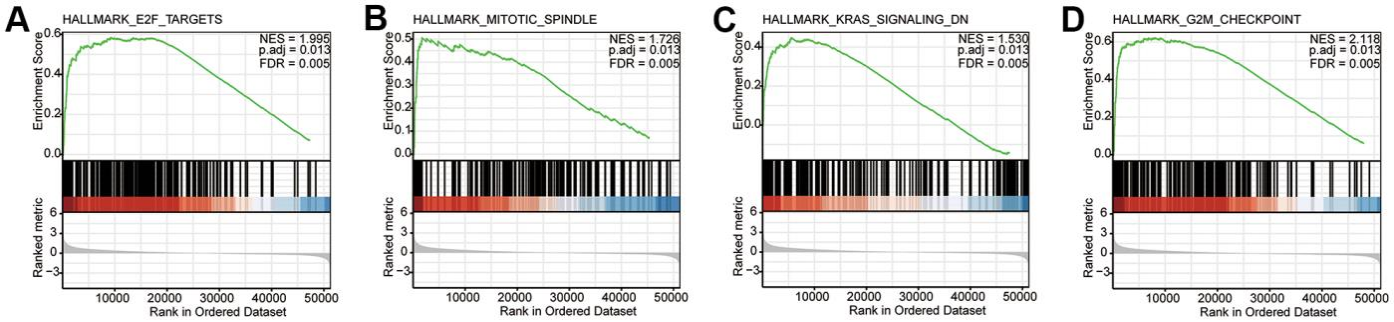
**CDCA3-related signaling pathways based on GSEA.** (**A**–**D**) Typical results of the GSEA for a single gene set. NES, normalized ES; *p*.adj, adjust *p* value; FDR, false discovery rate.

GSEA was used to identify signaling pathways involved in the difference between CDCA3-low expression group and CDCA3-high expression group in KIRP. Figure 4 shows typical results of the GSEA for a single gene set. The reference gene set was h.all.v7.0.symbols.gmt, the selected visualization data sets were HALLMARK_E2F_TARGETS (NES = 1.995, *p*.adj = 0.013, FDR = 0.005), HALLMARK_MITOTIC_SPINDLE (NES = 1.726, *p*.adj = 0.013, FDR = 0.005), HALLMARK_KRAS_SIGNALING_DN (NES = 1.530, *p*.adj = 0.013, FDR = 0.005), and HALLMARK_G2M_CHECKPOINT (NES = 2.118, *p*.adj = 0.013, FDR = 0.005). The data sets were significantly enriched in CDCA3-high expression group.

### Immune cell infiltration

**Figure 5:**
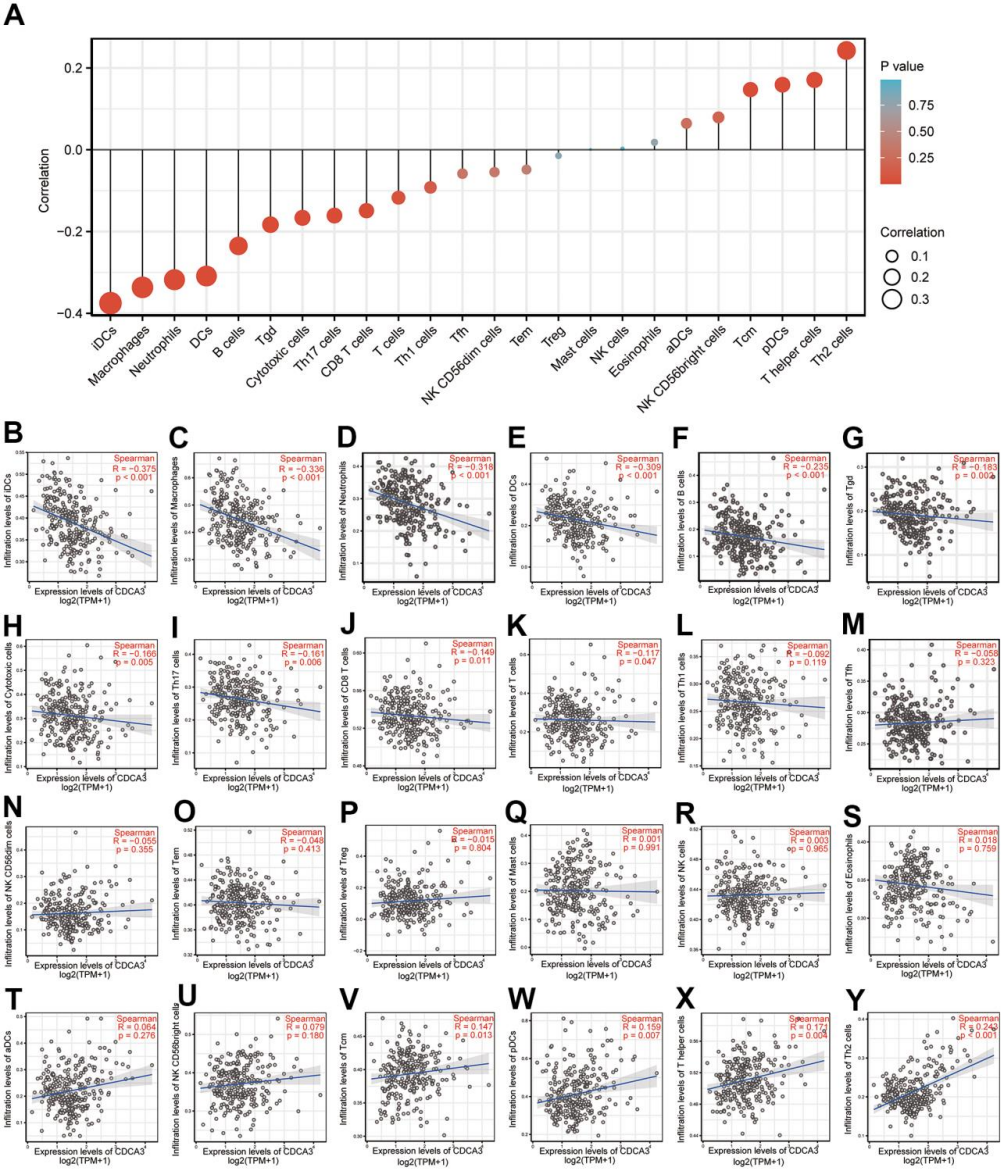
**Immune cell infiltration.** (**A**–**Y**) Spearman correlation analyses of the associations between the CDCA3 expression and the infiltration of 24 types of immune cells.

Spearman correlation analyses were performed to evaluate the associations between the CDCA3 expression and the infiltration of 24 types of immune cells quantified by ssGSEA in KIRP. We investigated whether the CDCA3 mRNA expression level correlated with immune infiltration levels in KIRP. The CDCA3 mRNA expression obviously related to frequencies of infiltrated iDCs, macrophages, neutrophils, DCs, B cells, Tgd, cytotoxic cells, Th17, CD8^+^ T cells, T cells, Tcm, pDCs, T helper cells and Th2 cells (Figure 5).

### PPI network construction

**Figure 6:**
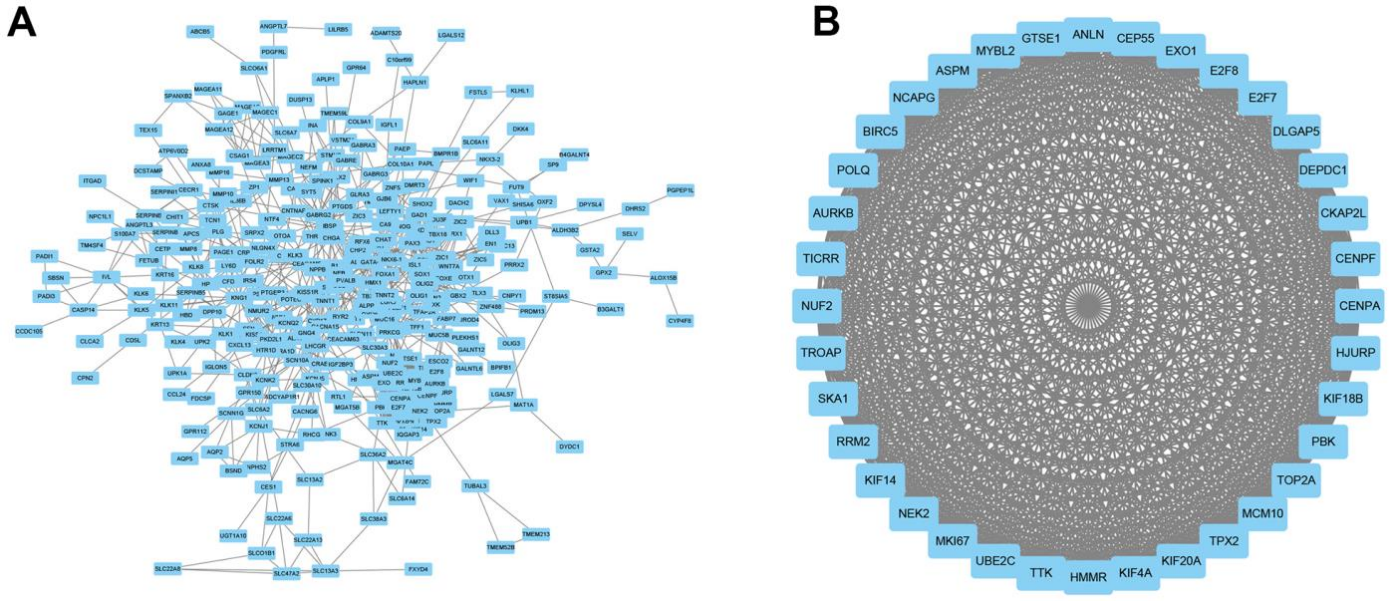
**PPI network construction.** (**A**) A PPI network was constructed using Cytoscape. (**B**) The most significant module was using MCODE of Cytoscape.

A PPI network was constructed using Cytoscape (Figure 6A) and the most significant module was selected using MCODE of Cytoscape (Figure 6B). The protein with the highest connectivity was identified as CENPF.

### Clinicopathological factors associated with CDCA3 in KIRP

**Figure 7:**
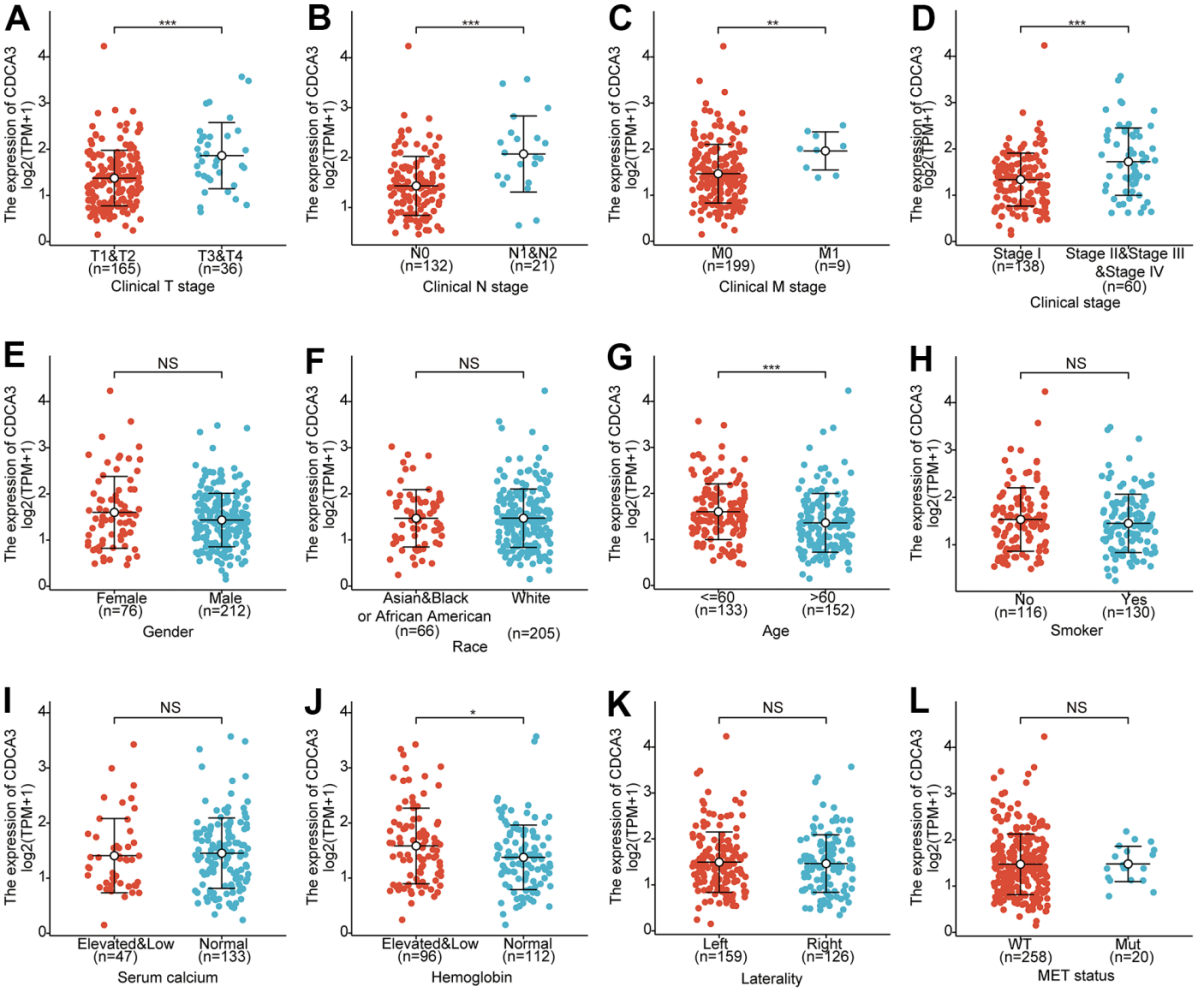
**Clinicopathological factors associated with CDCA3 in KIRP.** (**A**–**L**) CDCA3 mRNA expression with clinicopathological parameters of patients with KIRP, including clinical stage, gender, race, age, smoking status, serum calcium, hemoglobin, laterality and MET status. ns, *p* ≥ 0.05; *, *p* < 0.05; **, *p* < 0.01; ***, *p* < 0.001.

Next, the relationships between the CDCA3 mRNA expression with clinicopathological parameters of KIRP patients with KIRP were analyzed, including clinical stage, gender, race, age, smoking status, serum calcium, hemoglobin, laterality and MET status. As was shown in Figure 7, CDCA3 mRNA expressions levels remarkably associated with the clinical T stage, clinical N stage, clinical M stage, clinical stage, age and hemoglobin. No statistically significant relationships were observed between CDCA3 expression and gender, race, smoking status, serum calcium, laterality and MET. Consistent results were obtained using the chi-square test and Fisher’s exact test (Table 1).

**Table 1:** Clinicopathological factors associated with CDCA3 in KIRP.

**Characters**	**Level**	**Low expression of CDCA3**	**High expression of CDCA3**	**p**	**Test**
n		144	144		
Clinical T stage (%)	T1	78(75.0%)	61(62.9%)	0.002	exact
	T2	17(16.3%)	9(9.3%)		
	T3	9(8.7%)	26(26.8%)		
	T4	0(0.0%)	1(1.0%)		
Clinical N stage (%)	N0	70(95.9%)	62(77.5%)	0.001	exact
	N1	3(4.1%)	16(20.0%)		
	N2	0(0.0%)	2(2.5%)		
Clinical M stage (%)	M0	105(99.1%)	94(92.2%)	0.017	exact
	M1	1(0.9%)	8(7.8%)		
Clinical stage (%)	Stage I	79(76.7%)	59(62.1%)	<0.001	exact
	Stage II	16(15.5%)	5(5.3%)		
	Stage III	7(6.8%)	22(23.2%)		
	Stage IV	1(1.0%)	9(9.5%)		
Smoker (%)	No	54(45.0%)	62(49.2%)	0.525	exact
	Yes	66(55.0%)	64(50.8%)		
Gender (%)	Female	33(22.9%)	43(29.9%)	0.229	exact
	Male	111(77.1%)	101(70.1%)		
Race (%)	Asian	2(1.5%)	4(2.9%)	0.830	exact
	Black or African American	30(22.2%)	30(22.1%)		
	White	103(76.3%)	102(75.0%)		
Serum calcium (%)	Elevated	3(3.1%)	3(3.6%)	0.223	exact
	Low	27(27.8%)	14(16.9%)		
	Normal	67(69.1%)	66(79.5%)		
Hemoglobin (%)	Elevated	0(0.0%)	1(1.0%)	0.126	exact
	Low	44(40.7%)	51(51.0%)		
	Normal	64(59.3%)	48(48.0%)		
Laterality (%)	Left	80(56.3%)	79(55.2%)	0.905	exact
	Right	62(43.7%)	64(44.8%)		
MET status (%)	Mut	10(7.1%)	10(7.2%)	1.000	exact
	WT	130(92.9%)	128(92.8%)		
Age (%)	<=60	54(37.8%)	79(55.6%)	0.003	exact
	>60	89(62.2%)	63(44.4%)		
Age (median [IQR])		64.00[56.50,71.00]	60.00[52.25,67.00]	0.005	nonnorm

Collectively, our results showed that CDCA3 mRNA expression associated with some of the clinicopathological parameters of KIRP.

### ROC analysis

**Figure 8:**
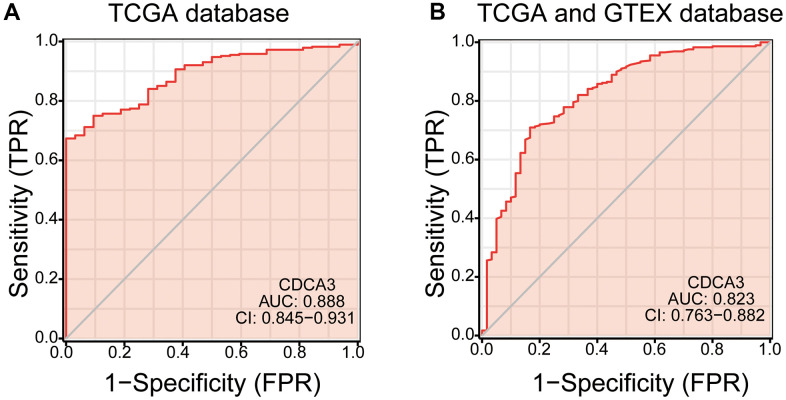
**ROC analysis.** (**A**, **B**) the diagnostic efficacy of CDCA3 for KIRP both the TCGA database and the TCGA combined with the GTEX database.

Performing ROC analysis, we determined the diagnostic efficacy of CDCA3 for KIRP. We found that the CDCA3 expression status could serve as a potential predictor for KIRP in both the TCGA database (AUC=0.888) and the TCGA combined with the GTEX database (AUC = 0.823) (Figure 8A, 8B).

### Logistic regression

The logistic regression method was used to analyze the relationships between clinicopathological characteristics and low or -high CDCA3 expression. CDCA3 expression significantly correlated with the clinical T stage (*p* < 0.001), clinical N stage (*p* = 0.003), clinical M stage (*p* = 0.041), and Clinical stage (p = 0.027) (Table 2).

**Table 2:** The relationships between clinicopathological characteristics and low or -high CDCA3 expression.

**Characteristics**	**Odds ratio in CDCA3 expression**	**Odds ratio(OR)**	**P value**
Clinical T stage (T3&T4 vs. T1&T2)	201	4.07(1.86-9.67)	<0.001
Clinical N stage (N1&N2 vs. N0)	153	6.77(2.16-29.90)	0.003
Clinical M stage (M1 vs. M0)	208	8.94(1.60-167.33)	0.041
Clinical stage (Stage II&Stage III&Stage IV vs. Stage I)	198	2.01(1.09-3.76)	0.027
Serum calcium (Elevated&Low vs. Normal)	180	0.58(0.29-1.13)	0.114
Hemoglobin (Elevated&Low vs. Normal)	208	1.58(0.91-2.74)	0.104
Laterality (Right vs. Left)	285	1.05(0.65-1.67)	0.853
MET status (Mut vs. WT)	278	1.02(0.40-2.56)	0.973

### Survival analyses

**Figure 9:**
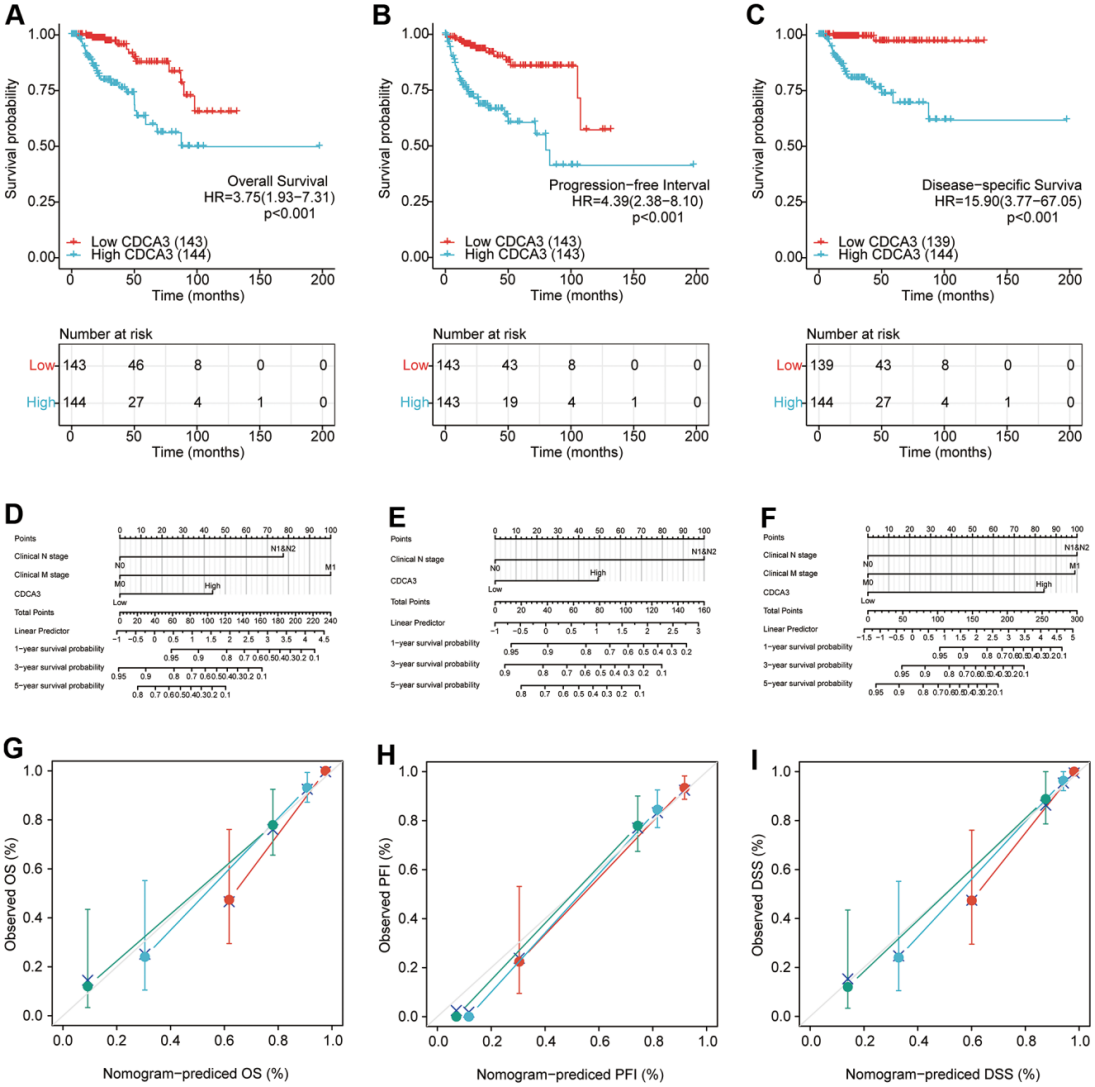
**Survival analyses.** (**A**–**C**)The prognostic value of CDCA3 with OS, PFI, DSS. (**D**–**F**) The nomogram of CDCA3 with OS, PFI, DSS. (**G**–**I**) The calibration curve of N stage (green), M stage (blue) and CDCA3 (red) with OS, PFI, DSS.

The Kaplan-Meier curves were generated to evaluate the prognostic value of CDCA3 with respect to the overall survival (OS), progression-free interval (PFI), and disease-specific survival (DSS) in CDCA3 expression subgroups in KIRP. High CDCA3 expression in KIRP associated with a worse OS (HR = 3.75(1.93-7.31), *p* < 0.001) (Figure 9A). Similar results were obtained in PFI analysis (HR = 4.39(2.38-8.10), *p* < 0.001) and DSS analysis (HR = 15.90(3.77-67.05), *p* < 0.001) analyses (Figure 9B, 9C).

A univariate analysis revealed that the clinical T stage, clinical N stage, clinical M stage, clinical stage, hemoglobin and CDCA3 expression were associated with a shorter OS. A multivariate analyses also revealed that the clinical N stage (*p* = 0.012), clinical M stage (*p* = 0.008), and CDCA3 expression (*p* = 0.017) were independent factors associated with a poor OS (Table 3).

**Table 3:** A univariate analysis and a multivariate analysis of OS.

**Characteristics**	**Total(N)**	**HR(95% CI)univariate analysis**	**P value univariate analysis**	**HR(95% CI)multivariate analysis**	**P valuemultivariate analysis**
Clinical T stage(T3&T4 vs. T1&T2)	201	4.687(2.292-9.587)	<0.001	0.517(0.103-2.610)	0.425
Clinical N stage(N1&N2 vs. N0)	153	10.637(4.972-22.755)	<0.001	8.218(1.595-42.346)	0.012
Clinical M stage(M1 vs. M0)	208	38.111(12.616-115.123)	<0.001	12.406(1.931-79.714)	0.008
Clinical stage(Stage II&III&IV vs.Stage I)	198	5.123(2.450-10.712)	<0.001	4.545(0.856-24.144)	0.076
Smoker(Yes vs. No)	245	0.564(0.298-1.069)	0.079	0.319(0.096-1.055)	0.061
Age (>60 vs. <=60)	285	0.956(0.525-1.738)	0.882		
Gender(Male vs. Female)	287	0.617(0.320-1.189)	0.149		
Race(White vs.Asian&Black or African American)	271	0.921(0.424-2.000)	0.834		
Serum calcium(Elevated&Low vs. Normal)	180	1.659(0.752-3.661)	0.21		
Hemoglobin(Elevated&Low vs. Normal)	208	4.381(1.877-10.223)	<0.001	1.958(0.589-6.502)	0.273
Laterality(Right vs. Left)	284	0.726(0.388-1.359)	0.317		
MET status(Mut vs. WT)	277	1.025(0.315-3.336)	0.967		
CDCA3(High vs. Low)	287	3.751(1.926-7.306)	<0.001	5.264(1.354-20.462)	0.017

Univariate analyses revealed that the clinical T stage (*p* < 0.001), clinical N stage (*p* < 0.001), clinical M stage (*p* < 0.001), clinical stage (*p* <0.001), gender (*p* = 0.026), hemoglobin (*p* = 0.039), and CDCA3 expression (*p* < 0.001) were associated with a worse PFI. A multivariate Cox regression further showed that the clinical N stage (*p* = 0.006) and CDCA3 expression (*p* = 0.017) were independent prognostic factors based on PFI (Table 4). Similar results were obtained in DSS analysis, indicating that clinical N stage (*p* = 0.012), clinical M stage (*p* = 0.008), and CDCA3 expression (*p* = 0.017) were independent factors associated with a poorer DSS (Table 5). Calibration curve were developed to evaluate the predictive accuracy of these predictors for OS, PFI, and DSS respectively. The independent predictors could predict the prognosis based on OS (C-index = 0.884(0.857-0.911)), PFI (C-index = 0.807 (0.773-0.841)), and DSS (C-index = 0.921(0.903-0.940)).

**Table 4:** A univariate analysis and a multivariate analysis of PFI.

**Characteristics**	**Total(N)**	**HR(95% CI)univariate analysis**	**P valueunivariate analysis**	**HR(95% CI)multivariate analysis**	**P valuemultivariate analysis**
Clinical T stage(T3&T4 vs. T1&T2)	200	7.383(3.906-13.955)	<0.001	1.565(0.420-5.825)	0.504
Clinical N stage(N1&N2 vs. N0)	152	17.022(8.265-35.057)	<0.001	7.079(1.774-28.254)	0.006
Clinical M stage(M1 vs. M0)	207	10.324(4.129-25.818)	<0.001	0.829(0.167-4.123)	0.819
Clinical stage(Stage II&III&IV vs. Stage I)	197	6.983(3.557-13.708)	<0.001	2.124(0.596-7.569)	0.245
Smoker(Yes vs. No)	244	1.230(0.708-2.139)	0.463		
Age(>60 vs. <=60)	284	0.820(0.483-1.391)	0.461		
Gender(Male vs. Female)	286	0.528(0.301-0.925)	0.026	2.035(0.591-7.009)	0.26
Race(White vs.Asian&Black or African American)	270	0.863(0.451-1.651)	0.657		
Serum calcium(Elevated&Low vs. Normal)	179	1.180(0.542-2.565)	0.677		
Hemoglobin(Elevated&Low vs. Normal)	207	1.976(1.035-3.772)	0.039	2.038(0.799-5.203)	0.136
Laterality(Right vs. Left)	283	0.770(0.443-1.339)	0.355		
MET status(Mut vs. WT)	276	1.158(0.416-3.221)	0.779		
CDCA3(High vs. Low)	286	4.388(2.376-8.105)	<0.001	3.293(1.241-8.740)	0.017

**Table 5:** A univariate analysis and a multivariate analysis of DSS.

**Characteristics**	**Total(N)**	**HR(95% CI)univariate analysis**	**P valueunivariate analysis**	**HR(95% CI)multivariate analysis**	**P valuemultivariate analysis**
Clinical T stage(T3&T4 vs. T1&T2)	200	8.926(3.806-20.932)	<0.001	0.428(0.077-2.375)	0.331
Clinical N stage(N1&N2 vs. N0)	153	19.162(7.687-47.767)	<0.001	7.003(1.299-37.743)	0.024
Clinical M stage(M1 vs. M0)	207	40.575(13.073-125.940)	<0.001	11.825(1.548-90.309)	0.017
Clinical stage(Stage II&III&IV vs. Stage I)	197	27.918(6.516-119.621)	<0.001	10.927(0.945-126.326)	0.056
Smoker(Yes vs. No)	242	0.610(0.284-1.310)	0.205		
Age(>60 vs. <=60)	281	0.447(0.206-0.969)	0.041	1.425(0.359-5.659)	0.615
Gender(Male vs. Female)	283	0.544(0.250-1.180)	0.123		
Race(White vs.Asian&Black or African American)	267	0.891(0.358-2.220)	0.805		
Serum calcium(Elevated&Low vs. Normal)	177	1.749(0.633-4.833)	0.281		
Hemoglobin(Elevated&Low vs. Normal)	205	3.174(1.204-8.368)	0.02	1.760(0.414-7.479)	0.444
Laterality(Right vs. Left)	280	0.508(0.223-1.155)	0.106		
MET status(Mut vs. WT)	273	0.508(0.069-3.754)	0.507		
CDCA3(High vs. Low)	283	15.895(3.768-67.047)	<0.001	5.264(1.093-25.343)	0.038

**Figure 10:**
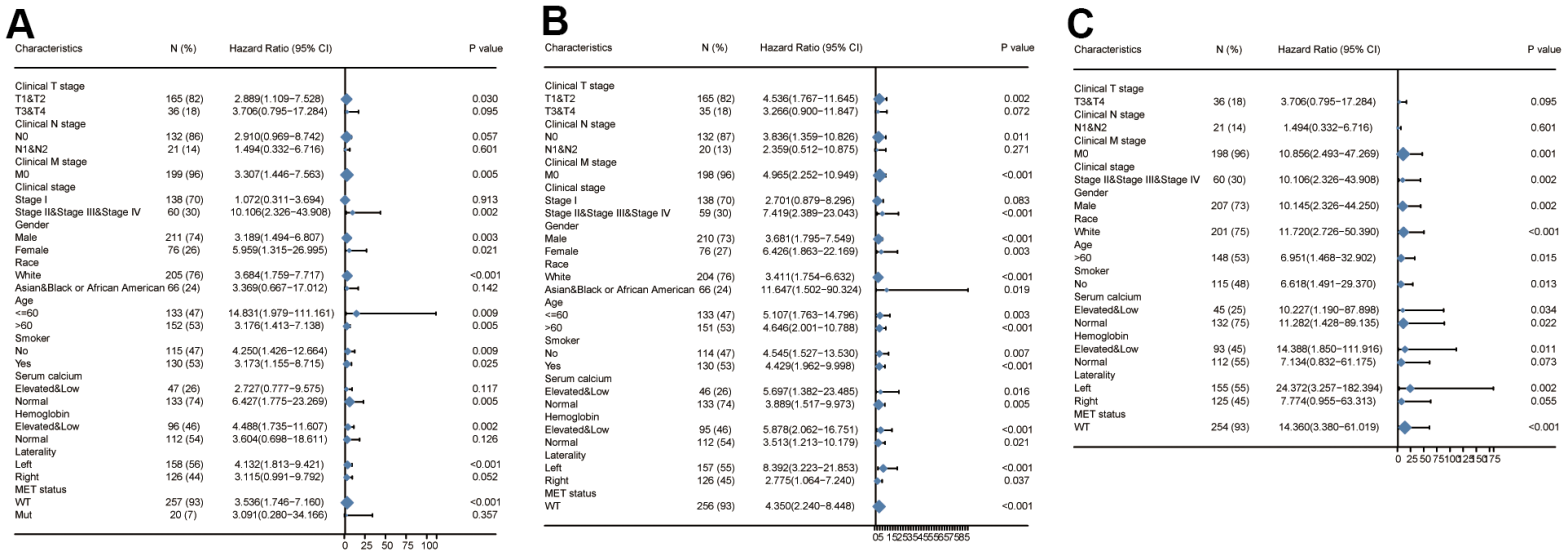
**Survival analysis of subgroups.** (**A**–**C**) Prognostic value of CDCA3 with OS, PFI, DSS of KIRP subgroup.

Finally, we analyzed the prognostic value of CDCA3 expression based on OS, PFI, and DSS in each clinicopathological subgroups of KIRP. As shown in Figure 10, the prognostic value of CDCA3 expression was statistically significant in the following subgroups: T1 and T2 for the clinical T stage (HR = 2.889(1.109-7.528), *p* = 0.030), and the M0 subgroup of the clinical M stage (HR = 3.307(1.446-7.563), *p* = 0.005), clinical stage II, stage III, and stage IV subgroups of clinical stage (HR = 10.106(2.326-43.908), *p* = 0.002), male subgroup (HR = 3.189(1.494-6.807), *p* = 0.003), female subgroup (HR = 5.959(1.315-26.995), *p* = 0.021), white subgroup of race (HR = 3.684(1.759-7.717), *p* < 0.001), age less than 60 years old subgroup (HR = 14.831 (1.979-111.161), *p* = 0.009), age over 60 years old subgroup (HR = 3.176 (1.413-7.138), *p* = 0.005), non-smoking subgroup (HR = 4.250 (1.426-12.664), *p* = 0.009), smoking subgroup (HR = 3.173 (1.155-8.715), *p* = 0.025), normal serum calcium subgroup (HR = 6.427(1.775-23.269), *p* = 0.005), elevated and low hemoglobin subgroup (HR = 4.488(1.735-11.607), *p* = 0.002), left laterality subgroup (HR = 4.132(1.813-9.421), *p* < 0.001), and wild type subgroup (HR = 3.536(1.746-7.160), *p* < 0.001). The analyses of the prognostic value of CDCA3 expression in each KIRP subgroup based on PFI and DSS also yielded similar results.

## DISCUSSION

KIRP accounts for approximately 10-20% of RCC cases. KIRP tends to occur in individuals over 50 years of age and affects more men than women, with a genetic predisposition. KIRP is typically discovered incidentally during physical examination. Some patients have typical clinical manifestations of RCC, such as hematuria, lumbago, and abdominal masses. The pathological features of KIRP are solid tumors in the renal cortex with clear boundaries. [8]. The prognosis of KIRP is better than that of KIRC, however, it is closely related to tumor stage or grade [27]. Compared with KIRC, KIRP grows slowly and is often enveloped. Distant metastasis and the infiltration of surrounding tissue are relatively rare. Most KIRP tumors have a low TNM stage.

Several cytokines, hormones, and proteins are involved in the development and progression of RCC and KIRP. Galectin-3 is widely expressed in RCC, and promotes the invasiveness, and suggestiveness via CXCR2, thereby affecting the occurrence and development of RCC [28]. Activation of p53 and HIF-1α promoted the transformation of RCC cells [29]. Peckova et al. found that most KIRP cells exhibit polysomy of chromosome 17 and chromosome 7 and expressed AMACR, OSCAR, CAM 5.2, HIF-2, and vimentin [30]. However, some type I KIRPs were accompanied by abnormalities of chromosomes 3, 12, 16, and 20 [31]. Mutations associated with KIRP, including MET mutations and mutations resulting in chromatin modifications, have been reported [5]. MET inhibitors could effectively improve the prognosis of metastatic KIRP [32, 33]. EpCAM has prognostic value in KIRP, and the overexpression of EpCAM in high-grade KIRP could be a useful indicator of prognosis [34]. However, compared with metastatic KIRP, TKI, and mTOR inhibitors are less effective in KIRP, with lower 5-year survival rates [35].

CDCA3, as a part of the skp1-cullin-f-box ubiquitin ligase complex, regulates the cell cycle by acting as an endogenous cell cycle inhibitor. CDCA3 participates in human physiological and pathological processes via regulating various downstream cytokines, hormones, and proteins. As shown in Figure 1A–1B, the expression of CDCA3 was up-regulated in a variety of tumor tissues. Several other studies have also shown that CDCA3 plays a significant role in the occurrence and development of tumors, including non-small cell lung cancer, prostate cancer, breast cancer, and KIRC [17]. The expression of CDCA3 in non-small cell lung cancer cells is significantly increased, and is closely related to a poor prognosis [18]. CDCA3 overexperssion promotes the proliferation of colorectal cancer cells, while knocking down CDCA3 expression *in vivo* and *in vitro* decreases the proliferation of colorectal cancer cells [36]. In particular, the inhibition of CDCA3 expression induces cell cycle arrest in colorectal cancer cells, thereby promoting cell apoptosis [37]. CDCA3 expression is increased in gastric cancer cells and is associated with a poor prognosis. CDCA3 overexpression *in vivo* and *in vitro* promotes the growth and colony formation ability of gastric cancer cells, while inhibiting CDCA3 expression mitigates these effects [38]. Furthermore, in gastric cancer CDCA3 expression is regulated by DNA methylation, and the binding activity of SP1 and the CDCA3 promoter is significantly up-regulated. Knockdown of SP1 downregulated CDCA3 expression, and the proliferation and invasion of gastric cancer cells is significantly inhibited [39]. In leukemia cell lines, miR-375 expression is down-regulated, and miR-375 inhibits CDCA3 expression by downregulating HOXB3 expression, thereby suppressing cell proliferation [38]. CDCA3 is overexpressed in bladder cancer and is related to prognosis [40] and its high expression is closely related to survival in breast cancer [41].

The prognosis value of CDCA3 in KIRP remains unclear and was the focus of this study. We observed that CDCA3 in KIRP tissues was significantly up-regulated compared to level in normal or paired paracancerous normal tissues (Figure 1C, 1D). Our results showed that compared to the levels in normal samples, CDCA3 mRNA expression in KIRP samples was significantly up-regulated based on the KIRP data from TCGA and the GTEx database. Moreover, 739 DEGs were identified between groups with low and high # expression. As shown in Figure 3A, CDCA3 and its related DEGs are involved several diverse biological processes, such as nuclear division and mitotic nuclear division. Qiu [42] reported that CDCA3 is involved in cell mitosis, validating our results. A GSEA indicated that CDCA3 is related to various gene sets, such as E2F targets, spindle formation during mitosis, KRAS signaling, and G2M checkpoints (Figure 4). E2F4 promotes proliferation and cell cycle progression in hepatocellular carcinoma cells by up-regulating CDCA3 expression [43]. Numerous studies have shown that CDCA3 is related to cell mitosis [36, 42]. These results confirmed the results of the GSEA in present study. We found that the infiltration of various immune cells was notably related to CDCA3 mRNA expression (Figure 5). Based on the TIMER database, Wang [44] found that CDCA3 is related to the infiltration of many immune cells in hepatocellular carcinoma. Immune cell infiltration is gaining increasing attention in tumor biology research, however, relatively few studies have explored the relationship between CDCA3 and immune cell infiltration. In our study, the PPI network was constructed by Cytoscape and the most significant module was selected by MCODE of Cytoscape (Figure 6). The highest connectivity was screened as CENPF, CENPA, KIF4A, UBE2C among others.

Studies have shown that the expression levels of CDCA3 and CENPF are correlated in esophageal carcinoma [45]. Levels of CDCA3, CENPF, CENPA and KIF4A are correlated in bladder cancer [40]. Our result showed that CDCA3 mRNA expressions remarkably correlated with the clinical T stage, clinical N stage, clinical M stage, clinical stage, age, and hemoglobin. Furthermore, the CDCA3 expression status had high diagnostic value in KIRP. Moreover, in a logistic regression analysis, CDCA3 was significantly correlated with clinical T stage, clinical N stage, clinical M stage, and clinical stage. High CDCA3 expression in KIRP was associated with a worse OS, PFI and DSS. Besides, univariate and multivariate analyses supported the prognostic value of CDCA3 based on OS, PFI and DSS in various subgroups of KIRP.

Despite presenting some credible data and experimental evidence, this study has some limitations. First, all the data were obtained from online databases and only *in silico* analyses were performed, further *in vivo* and *in vitro* studies are required to verify our results. Second, we found that CDCA3 was related to KIRP and could be used as a potential predictor of the prognosis. However, the underlying mechanisms by which CDCA3 regulates the occurrence and development of KIRP remains unclear. Further research studies to reveal the detailed mechanism underlying the relationship between CDCA3 and KIRP.

In conclusion, our results showed that CDCA3 is overexpressed in KIRP. The infiltration of various immune cells was notably related to CDCA3 mRNA expressions. Moreover, CDCA3 was significantly associated with the clinical T stage, clinical N stage, clinical M stage, clinical stage, age and hemoglobin in KIRP. Furthermore, high expression level of CDCA3 were significantly related to a shorter OS, PFI, and DSS in KIRP. Accordingly, CDCA3 is a potential target for the development of anti-KIRP therapeutics and an efficient prognostic marker for KIRP.
